# Efficacy of Chinese herbal medicine Jiangniaosuan formula for treatment of hyperuricemia: study protocol for a double-blinded non-inferiority randomized controlled clinical trial

**DOI:** 10.1186/s13063-021-05959-2

**Published:** 2022-01-03

**Authors:** Yafang Guo, Hong Lu, Jing Gan, Dongdong Li, Jiandong Gao, Changming Zhang

**Affiliations:** 1 Department of TCM Internal Medicine, Shanghai Pudong Hospital Affiliated to Fudan University, 2800 Gongwei Road, Shanghai, 201399 China; 2 Department of Nephrology, Shuguang Hospital Affiliated to Shanghai University of Traditional Chinese Medicine, 528 Zhangheng Road, Shanghai, 201203 China; 3 TCM Institute of Kidney Disease, Shanghai University of Traditional Chinese Medicine, 528 Zhangheng Road, Shanghai, 201203 China

**Keywords:** Chinese herbal medicine, Hyperuricemia, Protocol, Jiangniaosuan formula, Randomized controlled trial

## Abstract

**Background:**

Jiangniaosuan formula (JNSF) is commonly used in China for treating hyperuricemia, but there is little research-based evidence to support its use. This randomized controlled trial aims to assess the efficacy and safety of JNSF.

**Methods:**

A total of 72 patients with hyperuricemia will be selected and randomly assigned in a ratio of 1:2 to receive either Western medicine, i.e., febuxostat 40 mg (WG group; *n* = 24), or Chinese herbal medicine, i.e., Jiangniaosuan formula + febuxostat 20 mg (WJNSG group; *n* = 48). After 12 weeks, the WJNSG will be randomly divided into two groups of 24 patients each; one group (WJNSG; *n* = 24) still will receive febuxostat 20 mg + Jiangniaosuan formula, and the other group (JNSG; *n* = 24) will continue to receive Jiangniaosuan formula + placebo. Participants will be followed up at 4-week intervals. The primary outcome will be the change in serum uric acid level, and the secondary outcome will be the change in traditional Chinese medicine (TCM) syndrome scores. Serum creatinine, blood glucose, and insulin levels will also be measured.

**Discussion:**

We hypothesize that patients with hyperuricemia will benefit from JNSF. This study will provide evidence-based recommendations for clinicians.

**Dissemination:**

The results will be published in a peer-reviewed journal and disseminated by academic conferences. The datasets analyzed during the current study are available from the corresponding author on reasonable request.

**Trial registration:**

Chinese Clinical Trials Register ChiCTR2000041083. Registered on 3 May 2021. The protocol version number is V3.0, 20210301.

## Introduction

Rapid economic development and improvement in living standards over the past few decades have resulted in an increase in the prevalence of hyperuricemia across countries [1]. In China, the overall prevalence of hyperuricemia is 13.3%, with a higher prevalence reported in coastal areas (34.05%) and in men than in women (41.53% vs. 26.14%) [2–4].

Hyperuricemia results from an imbalance between the endogenous production of urate and its excretion. The most common mechanism is decreased excretion of urate. Studies show that hyperuricemia is an independent risk factor for chronic kidney disease (CKD), hypertension, cardiovascular and cerebrovascular diseases, and diabetes, as well as an independent predictor of premature death [5]. Hyperuricemia has been found to be significantly more common in individuals with above-normal serum triglyceride level [6] and to increase the risk for atherosclerosis as well as heart failure [7–9]. A study that followed up 177,570 patients for 25 years found that patients with high serum uric acid levels had 2.14 times higher risk for CKD than patients with normal serum uric acid [10]. Hyperuricemia has an independent adverse effect on renal function [11], and one prospective study from Japan that followed up individuals without CKD found that those with higher uric acid levels at baseline had a higher CKD incidence and a more rapid rate of decline in renal function [12].

In Western medicine, the commonly used serum uric acid–lowering drugs are allopurinol, febuxostat, and topiroxostat. Allopurinol, which is the most widely used, effectively lowers serum uric acid level in patients with or without gout, but is contraindicated in patients with renal dysfunction. Exfoliative dermatitis is the most serious adverse effect of allopurinol. Febuxostat is associated with a higher risk of cardiovascular events according to the US Food and Drug Administration. Meanwhile, topiroxostat, a novel nonpurine selective inhibitor of xanthine oxidoreductase that was developed as an alternative to conventional XOD inhibitors, has shown renoprotective effects, as demonstrated by the reduction of urinary albumin excretion, and thus can be used without dose reduction in patients with mild to moderate renal dysfunction [13, 14]. However, there is accumulating evidence that it may not be advisable in patients with concurrent chronic heart failure.

In China, compliance with uric acid–lowering treatment is poor. In one study that followed up patients for 4 weeks after a gout attack, only 20.7% adhered to the prescribed treatment, and only 18.9% achieved target serum uric acid levels [15]. A telephone follow-up of 539 patients with gout found that 40.9% of those in whom urate-lowering therapy was indicated had not taken the treatment in the past 12 months; medication adherence was only 21.6% [16]. Furthermore, a survey conducted during the current coronavirus pandemic found that a large proportion (41%) of gout patients were reluctant to go to hospitals for review [17]. Thus, it is obvious that hyperuricemia treatment is often less than satisfactory.

In China, Chinese herbal medicine (CHM) is often used to treat hyperuricemia. One popular medicine is Jiangniaosuan formula (JNSF), proposed by Professor Zheng Pingdong of Shuguang Hospital Affiliated to Shanghai University of Traditional Chinese Medicine (TCM). Over nearly 20 years of clinical observation, Professor Zheng found that phlegm dampness and blood stasis are the key to the pathogenesis of hyperuricemia. JNSF therefore was formulated to prevent blood stasis by stimulating blood circulation and to dissipate phlegm and descend the turbid. Over the past years, JNSF has been found to be safe and effective [18, 19].

### Objectives

The aim of this randomized clinical trial was to assess the efficacy and benefits of JNSF in the treatment of hyperuricemia. Based on the evaluation of the clinical efficacy of JNSF, we aim to explore its mechanism of action and provide more clinical evidence for the clinical treatment of hyperuricemia using JNSF.

## Methods

### Study design and participants

This single-center double-blinded non-inferiority randomized controlled trial is expected to last 3 years at the Shanghai Pudong Hospital affiliated to Fudan University in China. Individuals diagnosed with hyperuricemia will be recruited from the outpatient and inpatient departments of Shanghai Pudong Hospitals affiliated to Fudan University, China. Screening for eligibility will be conducted by clinicians not involved in the study. Patients who express willingness to participate and satisfy the inclusion and exclusion criteria listed below will be provided a detailed explanation—written in understandable language—regarding the purpose of the study and the methods that will be followed. All participants will provide their consent in writing.

A total of 72 individuals will be recruited and use the random number table randomly assigned in a 1:2 ratio to one of two treatment groups: a Western medicine group (WG; *n* = 24) and a Western medicine combine Chinese herbal medicine group (WJNSG; *n* = 48). Both groups will undergo a 24-week treatment. The protocol follows the Standard Protocol Items for Clinical Trials 2013 (SPIRIT 2013) [20].

### Diagnostic criteria for HUA

Participants are diagnosed with hyperuricemia which is defined as serum uric acid > 420 μmol/L on two repeated measurements (on different days)

### Diagnostic criteria for TCM syndrome differentiation

A patient should have at least two primary symptoms and two secondary symptoms, consistent with tongue and pulse examination which is diagnostic of phlegm and blood stasis syndrome.

### Inclusion criteria

Hyperuricemia is defined as serum uric acid > 420 μmol/L on two repeated measurements (on different days):

- Age 18–80 years
- No history of acute gouty arthritis or of treatment with any uric acid–lowering drugs in the preceding 2 weeks
- Meet TCM diagnostic criteria combined phlegm and blood stasis syndrome [21]

### Exclusion criteria

- History of cardiovascular disease
- Serious respiratory, digestive, hematological, and liver disease; malignancy; infectious disease; mental disorder
- History of allergy to TCM
- Pregnancy or lactation
- Not expected to comply with prescribed medications or to attend regular follow-up
- History of participation in another clinical trial in the previous 2 weeks
- Refusal to sign the consent form

### Participant elimination criteria

Patients will be removed/withdrawn from the study if they meet any of the following criteria:

- Patients who are not follow up on time or fail to follow up the protocol
- The creatinine level 30% higher than baseline, drug dose change in response to harms
- The alanine transaminase (ALT) or aspartate transaminase (AST) level 3 times higher than normal
- Patients with serious adverse events, complications, or specific physiological changes, who are not suitable to continue the trial
- Patients with insufficient evidence, failing to determine the effectiveness of the trial, request discontinuing

### Interventions

All of the groups will be treated with test drugs or placebo together with basic treatment.

Basic treatment: Avoid strenuous exercise, low purine diet, drink more water, control blood pressure, blood sugar

Test drugs:

WG: basic treatment + febuxostat 40 mg once daily; febuxostat (trade name: You litong in Chinese pinyin) is manufactured by Jiangsu Wanbang biochemical medicine group Co., Ltd.

WJNSG: basic treatment + febuxostat 20 mg once daily + Jiangniaosuan formula (200 mL of the decoction) two times a day orally. Constituents of Jiangniaosuan formula are shown in Table 1.

**Table 1:** Constituents of Jiangniaosuan formula

Chinese name	Scientific name	Dosage (g)	Purported effect
王不留行子 (Wang bu liu xing zi)	*Vaccaria hispanica* (Mill.) Rauschert	10	Promotes dampness and reduces jaundice
白芥子 (Bai jie zi)	*Semen sinapis*	10	Resolves phlegm and dissipates stagnation
车前子(Che qian zi)	*Plantaginis semen*	10	Clearing dampness and eliminates phlegm
粉萆薢 (Fen bi xie)	*Dioscorea hypoglauca* Palibin	10	Removes dampness and turbidity, dispels wind, and relieves arthralgia
生山楂 (Sheng shan zha)	*Crataegus pinnatifida* Bunge	10	Promotes circulation and prevents stasis
威灵仙 (Wei ling xian)	*Clematis chinensis* Osbeck	15	Dispels wind and dampness, activates meridians, and relieves pain
制大黄 (Zhi da huang)	*Rheum palmatum* L	10	Purges heat and promotes defecation, cools and detoxifies blood, relieves blood stasis, and dredges meridians

JNSG: basic treatment + febuxostat placebo daily + Jiangniaosuan formula (200-mL decoction) two times a day orally. JNSF are manufactured by Shanghai Kangqiao Chinese Medicine Tablet Co. Febuxostat placebo taste and packaging same as febuxostat, which are manufactured by Jiangsu Wanbang biochemical medicine group Co., Ltd.

### Groups

The total treatment process will be divided into two stages:

*Stage 1*: WG patients (*n* = 24) will receive febuxostat, while the WJNSG (*n* = 48) patients will receive febuxostat 20 mg plus Jiangniaosuan formula. After 12 weeks of this treatment, patients will enter the second stage of treatment.

*Stage 2*: WJNSG patients (*n* = 48) will be randomly divided into two equal subgroups: WJNSG (*n* = 24) will continue to receive febuxostat 20 mg and Jiangniaosuan formula, while the other subgroup (JNSG) will receive Jiangniaosuan formula and febuxostat placebo. The second stage will last for 12 weeks too.

**Fig. 1:**
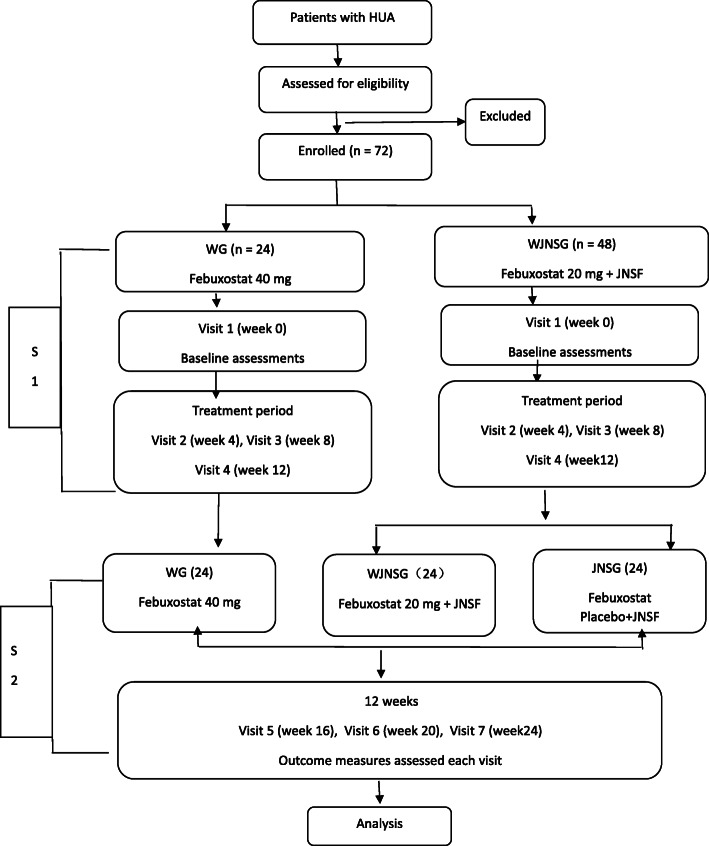
Study flow diagram. HUA hyperuricemia, WG Western medicine group, WJNSG Western medicine + Chinese herbal medicine group, JNSG Jiangniaosuan formula group, S stage

The study flow chart is shown in Fig. 1.

### Recruitment

Hyperuricemia patients in the Department of Integrated Traditional Chinese and Western Medicine and the nephrology department of Shanghai Pudong Hospital could receive detailed details of this study. The doctors, nurses, and careworkers for patients in the ward were informed. They will encourage patients to participate.

### Follow-up

During the 24 weeks of treatment, the participants will be asked to visit the clinic every 4 weeks. At each follow-up visit, blood and urine will be examined and the TCM symptom score will be calculated. The whole process will be supervised by 2 different supervisors. Strategies to improve adherence to treatment include series of diet courses on uric acid–lowering and certain transportation subsidies, which will promote retention and completion of follow-up assessments. Other uric acid–lowering treatments during the trial are not allowed.

### Outcome assessment

Participants will be assessed at seven time points: at baseline and at 4 weeks, 8 weeks, 12 weeks, 16 weeks, 20 weeks, and 24 weeks (Fig. 1).

#### Primary outcomes

The primary outcomes of this study will be the serum levels of uric acid.

#### Secondary outcome measures

Secondary outcomes include TCM symptom improvement, Scr, BUN, eGFR, 24 h UUA, fasting insulin, TG, TC, HDL, and LDL.

CHM is prescribed by TCM physicians according to patients’ symptoms and signs, tongue, manifestations, and pulse condition. The TCM symptom scoring form is designed to assess the internal accumulation of phlegm and blood stasis [21]. These symptoms are scored on an ordinal scale of absent, mild, moderate, and severe. The scoring tool will be completed by the assessing TCM physician, and the patient’s pulse and tongue appearance will be assessed from the TCM perspective. Information collected from the questionnaire will be used to assess the response to JNSG. Each symptom or sign will be given a score (Table 2), and the total score will be recorded. The change of score can calculate an efficacy indicator (EI) for the treatment efficacy.

**Table 2:** SPIRIT table: measurement items and content for the schedule of enrollment, interventions, and assessments

Items	Study period							
	Screening	Baseline	Treatment period					
	Visit 1	Visit 2	Visit 3	Visit 4	Visit 5	Visit 6	Visit 7	Visit 8
	−1w	0w	4w	8w	12w	16w	20w	24w
**Enrolment**								
Inclusion criteria	√							
Exclusion criteria	**√**							
Informed consent	**√**							
Randomization	**√**							
**Interventions**								
WG (*n* = 24)		**√**	**√**	**√**	**√**	√	√	√
WJNSG (*n* = 48/24)		√	**√**	**√**	**√**	√	√	√
JNSG (*n* = 24)						√	√	√
**General**								
Demographic data	√							
Symptoms and signs	√							
Past medical history and treatment history	√							
Complications and drug combination	√							
Physical examination (BP, P, R, T)	√	√	√	√	√	√		√
**Vital signs**								
TCM symptoms and signs	√	√	√	√	√	√	√	√
Renal function (SUA, Scr, BUN)	√	√	√	√	√	√	√	√
Laboratory index (fasting insulin, TG, TC, HDL, LDL, blood electrolytes)		√	√	√	√	√	√	√
24-h uric acid in urine		√	√	√	√	√	√	√
**Safety data**								
Routine urinalysis	√	√	√	√	√	√	√	√
Blood routine	√	√	√		√	√		√
Liver function (ALT, AST, γ-GT)	√	√	√		√	√		√
Electrocardiogram	√	√			√			√
Stool routine	√	√			√			√
**Other data**								
Causes of withdrawal or dropout		√	√	√	√	√	√	√
Safety evaluation		√	√	√	√	√	√	√
Adverse events		√	√	√	√	√	√	√
Analysis		√	√	√	√	√	√	√

*BP* blood pressure, *P* pulse, *R* breathing, *T* temperature, *Scr* serum creatinine, *BUN* blood urea nitrogen, *SUA* serum uric acid, *TG* blood triglyceride, *TC* serum total cholesterol, *HDL* high-density lipoprotein, *LDL* low-density lipoprotein. √ *n* = 48

EI = (Total symptom score at baseline − Total symptom score post−treatment)/Total symptom score at baseline × 100%

The degree of symptom improvement will be divided into four categories: full recovery (EI ≥90%), good recovery (90% > EI ≥ 70%), modest recovery (70% > EI ≥ 30%), and no recovery (EI < 30%).

### Data collection

Both cohorts undergo a 24-week treatment. Case-report forms, prepared prior to study commencement, will be used by the researchers to collect a range of information. Patients who fail to visit the clinic for follow-up will be contacted over the phone (Table 2). Each visit allows a window of 3 days.

#### Fundamental data

① Demographic data: age, sex, nationality, occupation, and so on

② Physical examination: breathing, body temperature, pulse, blood pressure, weight, waist-hip ratio, tongue coating, pulse, and so on

③ General clinical data: history of comorbidity, allergy, family history, and so on

Laboratory index: SUA, 24-h urine uric acid (24hUUA), Scr, BUN, fasting insulin, TG, TC, HDL, and LDL.

Safety indicators: blood routine, urine routine, stool routine, liver and kidney function, and blood electrolyte electrocardiogram

### Safety assessment

For safety monitoring and assessment, the following biological indicators will be assessed at each follow-up visit: blood routine, liver function (aspartate transaminase, alanine transaminase), renal function (blood urea nitrogen, serum creatinine), stool routine, and electrocardiogram.

Any unexpected symptoms or signs or feelings of discomfort will be recorded as adverse events (AEs). For each adverse event, the duration (starting date, ending date), severity, relationship to the trial, and potential to trigger patient dropout from the study will be recorded. Patients experiencing an adverse event will be monitored until it resolves.

When adverse events are found, the doctor reported to the project leader to assist DSMC in determining the treatment plan according to the patient’s condition, and a follow-up investigation should be carried out for the cases of drug withdrawal due to adverse reactions. All of the interventions should be recorded in detail. In case of serious adverse events, the researcher shall immediately take protective measures for the subjects and report to the sponsor, ethics committee, and DSMC within 24 h. Any adverse reactions are actively treated and followed up until fully improved.

All blood specimens after the test will be collected and stored in a specific −80 °C refrigerator of Pudong Hospital, which is kept by special personnel of the clinical laboratory. The later test can be applied. Until the experiment is complete, sterilization is performed.

### Data and safety monitoring

An independent Data Safety and Monitoring Committee (DSMC) includes statisticians, cardiologist, and so on. All relevant investigators will receive a 2-h training before the trial begins. They will monitor the whole trial process. They will join an online organization WeChat group, and they provide day-to-day support and coordinate works smoothly every day. They will meet once every month to monitor the safety of the trial and detect any difficulties or errors. It is independent from the sponsor and competing interests. All of the patients’ data will be stored in a password-protected Excel file. Healthcare inspectors, auditors, monitors, and members of the medical ethical commission might be allowed to access the database and biological specimens as required by the law. All data collected using the case-report forms will be submitted to an online clinical research data capture computer system developed by the research group. Two researchers are in charge of the database data. The data entry adopts a double-person verification system, one-person entry and one-person verification to ensure that the data are correct. Only the two data researchers participate in data recruitment or intervention.

### Protocol amendments

All trial team members join an online research working WeChat group. If there are any plans and notifications to change the study, the final protocol will be announced in the working WeChat group to ensure that everyone receives a notification. All members of the research team, the organizer, the doctors, the nurses, and the data researchers will perform the latest protocol after receiving the eventual modifications of the study. At the same time, the researchers will update the protocol in the clinical trial registry.

### Confidentiality

Personal information will be protected carefully from the beginning of the recruitment process. The patient’s information will be graded on a notebook from the first day of enrollment. The personal information including name, age, gender, contact family address, and emergency contact person will be kept in a separate notebook. When registered to the computer, each person will anonymously be a number, and the information will be registered to a folder with a password, and the name of the patient will be deleted. At last, we will use the summary data instead of the personal data in order to protect the research’s privacy, even the anonymized individual trial data will not be shared with other researchers.

### Sample size calculation

To detect treatment response rates of 93.3% and 63.3%, respectively, in the JNSF group and the control group with a significance level of 5% and power of 80%, we would need to have 30 patients in each group (total 60 patients). Assuming that there might be a 10% loss to follow-up or early withdrawal from the study, we decided to enroll 72 patients, with 24 patients in each group. These assumptions have been based on data of Zheng PD [18].

### Randomization

The randomization list was generated using a random number table by a statistician using SPSS. A statistician unrelated to this trial will use the random number table assigning the patients. After screening, if the patient agrees to participate in the trial and signs the informed consent form, he/she will receive a random number from the physician. In stage 1, 72 patients will be randomly divided into WG and WJNSG in a ratio of 1:2. In stage 2, after a 4-week treatment, the WJNSG will be randomly divided into WJNSG and JNSG in a ratio of 1:1. The sequence will be sealed in the opaque envelope. Only the statistician who generated the randomization is aware of the group assignments.

### Blinding

This clinical trial will be double-blinded, from stage 1 to stage 2 of the study. Physicians and patients were all blinded for the allocation. Only the statistician knows the allocation.

If an emergency occurs, the study designer will ask an independent physician to unblind the patient. The incident will be recorded and reported to the independent safety monitoring board. When all the data analysis is finished, the results will be released.

### Statistical analysis

Data analysis will be performed by a statistician who does not know the groupings in accordance with the specified statistical plan. The statistician comes from the Experimental Center of Shanghai Pudong Hospital, Shanghai, China. SPSS 22.0 (IBM Corp., Armonk, NY, USA) will be used to analyze the experimental data. Baseline differences between groups, gender, and age will be assessed with the *χ*^2^ test or Student’s *t*-test. Continuous variables will be presented as means ± standard deviations or medians, and differences in such variables will be analyzed using an independent *t*-test. Comparisons between the groups and within groups will be conducted using analysis of covariance (ANCOVA), with the baseline as the covariate. For efficacy analysis, repeated-measures analysis of variance (ANOVA) will be used to analyze the changes in scores from baseline to the endpoint of treatment. Within each group, differences will be assessed with the paired *t*-test for normally distributed data and the Wilcoxon signed-rank test for non-normally distributed data. For analysis of the final dataset, missing data will be filled in using the last-observation-carried-forward approach. *P* < 0.05 will be considered significant. No additional analyses are scheduled.

## Discussion

Hyperuricemia is currently a major public health problem in China, where TCM is often used for symptom relief and quality of life improvement. This study aims to examine the efficacy and safety of CHM in the management of hyperuricemia. Outcomes assessed during follow-up will include mortality rate and adverse events. These outcomes can be easily assessed with only a few questions. The physicians who will be performing the follow-up are familiar with the patients and are experienced interviewers. Therefore, the quality of the follow-up is guaranteed.

Western medicine is widely used for the treatment of hyperuricemia, but many side effects are reported. In China, the combination of TCM and Western medicine has been successfully used for the treatment of hyperuricemia for many years. However, there is little research evidence to support the efficacy of TCM and no clarity on its mechanism of action in hyperuricemia.

JNSF is a herbal preparation formulated by Professor Pingdong Zheng, a reputed TCM nephropathy expert. Professor Zheng believes that hyperuricemia results from impaired function of the spleen and stomach due to improper diet. In TCM theory, the spleen and stomach govern the transportation and transformation of water and nutrients. An improper diet adversely affects the physiological functioning of the spleen and stomach; the consequent accumulation of dampness and production of phlegm inhibits qi movement and leads to blood stasis, the end result being a syndrome of phlegm–dampness–blood stasis. Professor Zheng sought a way to resolve dampness, fortify the spleen, and stimulate blood circulation and came up with JNSF.

JNSF contains seven herbal ingredients (Table 1). All seven herbal constituents can drain dampness, resolve phlegm, activate blood, and dredge collaterals. The potential mechanism of JNSF in the treatment of hyperuricemia needs to be further explored. Some of the herbs in JNSF have proven pharmacological effects. Emodin has been shown to reduce serum uric acid level in rat models of hyperuricemia by inhibiting xanthine oxidase (XOD) generation [22]. *Rheum palmatum* L can reduce acute gout arthritis symptoms [23]. Calycosin and ethanol extract of Semen plantaginis can inhibit the activity of liver XOD in hyperuricemic mice, reduce the generation of oxygen free radicals, reduce kidney damage, and promote renal uric acid excretion [24]. Experimental studies have found that *Clematis chinensis* Osbeck can significantly reduce the deposition of urate crystals in renal tubulointerstitium in rats with uric acid nephropathy [25]. Total flavonoids of hawthorn leaves can regulate the activity of XOD and thus decrease serum uric acid level, raise nitric oxide level, and reduce endothelin level, and thereby minimize uric acid–induced damage to vascular endothelial cells [26]. Its main components steroid saponins have been shown to have strong uric acid–lowering, kidney protective, and immunomodulatory effects [27–29 ]. JNSF has been used for more than 20 years in China and has proved to be safe and well tolerated. However, there is still a dearth of research-based evidence on the benefits of TCM in hyperuricemia. The results of this trial will be useful for researchers, practitioners, and patients.

This study has some limitations. First, this study will focus on short-term outcomes and, therefore, we will be unable to comment on the downstream effects of the intervention. Second, this will be a single-center study; the results may not be applicable to other hospitals or regions. Third, JNSF is a composite preparation; which components provide the therapeutic effect will have to be clarified in future research. Fourth, this study is single-blinded which is a source of bias.

## Trial status

The study protocol and research strategy were developed from January 2021 to December 2022. Participant enrollment will occur from March 2022 to December 2022, and follow-up and data analysis will be conducted from December 2022 to December 2023. The protocol version number is V3.0, 20210301.

## Data Availability

Does not apply.

## Abbreviations

TCM: Traditional Chinese medicine
CHM: Chinese herbal medicine
CKD: Chronic kidney disease
JNSF: Jiangniaosuan formula
XOD: Xanthine oxidase
Scr: Serum creatinine
BUN: Blood urea nitrogen
SUA: Serum uric acid
TG: Blood triglyceride
TC: Serum total cholesterol
HDL: High-density lipoprotein
LDL: Low-density lipoprotein
UUA: Uric acid in urine
